# Recent Advances in Molecular Diagnostics and Targeted Therapy of Myeloproliferative Neoplasms

**DOI:** 10.3390/cancers13205035

**Published:** 2021-10-09

**Authors:** Simona Stivala, Sara C. Meyer

**Affiliations:** 1 Department of Biomedicine, University Hospital Basel and University of Basel, 4031 Basel, Switzerland; simona.stivala@unibas.ch; 2 Division of Hematology, University Hospital Basel, 4031 Basel, Switzerland

**Keywords:** myeloproliferative neoplasms, JAK2, gene mutations, next generation sequencing, epigenetics, JAK2 inhibition, resistance, targeted therapy

## Abstract

**Simple Summary:**

Myeloproliferative neoplasms (MPN) are clonal hematologic malignancies with dysregulated myeloid blood cell production driven by JAK2, calreticulin, and MPL gene mutations. Technological advances have revealed a heterogeneous genomic landscape with additional mutations mainly in epigenetic regulators and splicing factors, which are of diagnostic and prognostic value and may inform treatment decisions. Thus, genetic testing has become an integral part of the state-of-the-art work-up for MPN. The finding that JAK2, CALR, and MPL mutations activate JAK2 signaling has promoted the development of targeted JAK2 inhibitor therapies. However, their disease-modifying potential remains limited and investigations of additional molecular vulnerabilities in MPN are imperative to advance the development of new therapeutic options. Here, we summarize the current insights into the genetic basis of MPN, its use as diagnostic and prognostic tool in clinical settings, and recent advances in targeted therapies for MPN.

**Abstract:**

Somatic mutations in JAK2, calreticulin, and MPL genes drive myeloproliferative neoplasms (MPN), and recent technological advances have revealed a heterogeneous genomic landscape with additional mutations in MPN. These mainly affect genes involved in epigenetic regulation and splicing and are of diagnostic and prognostic value, predicting the risk of progression and informing decisions on therapeutic management. Thus, genetic testing has become an integral part of the current state-of-the-art laboratory work-up for MPN patients and has been implemented in current guidelines for disease classification, tools for prognostic risk assessment, and recommendations for therapy. The finding that JAK2, CALR, and MPL driver mutations activate JAK2 signaling has provided a rational basis for the development of targeted JAK2 inhibitor therapies and has fueled their translation into clinical practice. However, the disease-modifying potential of JAK2 inhibitors remains limited and is further impeded by loss of therapeutic responses in a substantial proportion of patients over time. Therefore, the investigation of additional molecular vulnerabilities involved in MPN pathogenesis is imperative to advance the development of new therapeutic options. Combination of novel compounds with JAK2 inhibitors are of specific interest to enhance therapeutic efficacy of molecularly targeted treatment approaches. Here, we summarize the current insights into the genetic basis of MPN, its use as a diagnostic and prognostic tool in clinical settings, and the most recent advances in targeted therapies for MPN.

## 1. Introduction

Myeloproliferative neoplasms (MPN) are clonal hematologic malignancies, which are characterized by an excessive output of mature myeloid blood cells and arise from mutant hematopoietic stem/progenitor cells [1]. MPN were first recognized as an entity by William Dameshek in 1951, who proposed that dysregulated myeloid blood cell proliferation was driven by a yet undefined stimulus [2]. In polycythemia vera (PV), this primarily becomes manifest as erythrocytosis, while essential thrombocythemia (ET) is characterized by thrombocytosis, and primary myelofibrosis (PMF) shows increased megakaryocytes along with progressive fibrosis of the bone marrow. Thrombohemorrhagic complications account in large parts for morbidity and mortality of MPN. Of note, all three forms may progress to acute myeloid leukemia mostly with high risk genetic features and dismal prognosis [1]. It was not until 2005 that technological advances in DNA sequencing led to the discovery of the *JAK2*V617F mutation [3,4,5,6], the most frequent driver mutation in MPN, followed by the identification of additional driver mutations in exon 12 of *JAK2* [7], the thrombopoietin receptor *MPL* [8,9] and calreticulin (*CALR*) genes [10,11]. With the increased accessibility of next-generation sequencing (NGS) approaches to large patient cohorts, we have recently gained more detailed insights into the heterogeneous genetic landscape of MPN and how additional mutations, together and in interplay with the so-called driver mutations, play significant roles in determining the clinical phenotype and course of MPN [12,13,14]. This has impacted on the way we diagnose MPN, as we increasingly rely on genetic/genomic information in addition to typical bone marrow morphology findings [15,16,17,18]. It has also enabled the development of small molecule tyrosine kinase inhibitors targeting JAK2 with clinical benefits for MPN patients [19,20,21,22,23,24,25]. In this review, we discuss the current knowledge regarding the mutational landscape of MPN and its diagnostic and prognostic value, as well as novel therapeutic options rationally designed based on the recent molecular insights and potential future targets for MPN therapy.

## 2. Genetic Landscape of Myeloproliferative Neoplasms

### 2.1. Somatic Driver Mutations

Myeloproliferative neoplasms arise from hematopoietic stem/progenitor cells with somatic gene mutations enabling clonal expansion. Limiting dilution experiments have demonstrated that a single hematopoietic stem/progenitor cell harboring the *JAK2*V617F mutation is sufficient to drive the emergence of an MPN phenotype in mouse models [26]. Thus, *JAK2*V617F and analogously mutations in *CALR* or *MPL* genes, which per se can drive outgrowth of a mutant cell clone and induce an MPN phenotype, are considered as driver mutations. They have been incorporated as a major criterion into the World Health Organization (WHO) diagnostic criteria of MPN and represent standard laboratory testing for patients with suspected MPN [15].

**Figure 1:**
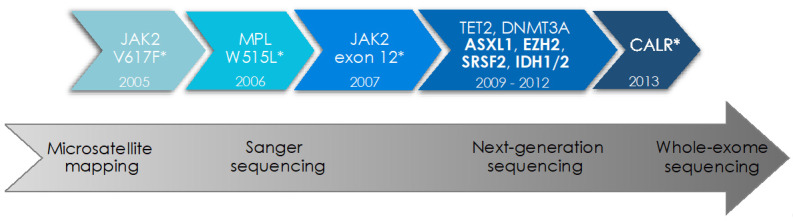
Identification of driver mutations and additional somatic mutations in myeloproliferative neoplasms by modern sequencing technologies. The advent of modern sequencing technologies over the last two decades has led to the characterization of the genetic landscape in myeloproliferative neoplasms (MPN), including driver mutations (*) in JAK2, MPL, and CALR genes as well as concomitant somatic mutations in cancer genes frequently mutated in myeloid malignancies. Mutations in the genes highlighted in bold are considered of “high molecular risk” (HMR) given their adverse prognostic impact. The year of discovery of specific mutations is indicated as well as the development of sequencing methodologies over time.

The valine to phenylalanine substitution at position 617 of the JAK2 protein is located in the pseudokinase domain of JAK2 and was the first driver mutation to be discovered in 2005 (Figure 1) [3,4,5,6]. JAK2 is an intracellular tyrosine kinase essential for intracellular signaling downstream of hematopoietic cytokine receptors including thrombopoietin (TPO), erythropoietin (EPO), and granulocyte-colony stimulating factor (G-CSF) receptors [27,28]. The *JAK2*V617F mutation impedes the inhibitory effect of the pseudokinase on the kinase domain and results in constitutive activation of JAK2 and its downstream effectors, including the signal transducer and activator of transcription (STAT) proteins, the phosphatidylinositol 3-kinase (PI3K), and the mitogen-activated protein kinase (MAPK) pathways promoting proliferation and survival of myeloid cells [29,30]. *JAK2*V617F is the most frequent MPN driver mutation detected in 95% of PV and 50–60% of ET and PMF patients [1,31]. It is still not completely understood how *JAK2*V617F gives rise to the differential clinical phenotypes of PV, ET, and MF. *JAK2*V617F mutant allele frequency and type and sequence of additional mutations in hematopoietic stem/progenitor cell sub-clones seem to influence the specific disease manifestations [32,33,34,35]. JAK2 exon 12 mutations are identified in ~4% of PV patients and lead to a similar constitutive activation of JAK-STAT signaling. While two thirds of JAK2 exon 12 mutated patients present with isolated erythrocytosis in the absence of concomitant leuko- or thrombocytosis, the clinical course is analogous to JAK2V617F mutant PV [36].

Calreticulin (*CALR*) is a chaperone protein in the endoplasmic reticulum (ER) involved in calcium homeostasis and protein folding. Mutations of the calreticulin gene are detected in 30–40% of ET and PMF patients [10,11]. They were first identified in 2013 with a 52 bp deletion (type 1 mutation) or 5 bp insertion (type 2 mutation) in exon 9 of *CALR* being most prevalent, while more than 35 mutations were described overall. It has been shown that the various *CALR* mutations all result in one-base pair frameshift leading to a novel C-terminus of the protein. Mutant CALR induces aberrant activation of MPL by binding to the receptor both in the ER and on the cell surface [37,38,39]. Of note, *CALR* mutations associate with a favorable prognosis and a lower risk for leukemic transformation compared to *JAK2*V617F mutated MPN, which impacts on therapeutic management [40,41,42].

*MPL* gene mutations affecting the thrombopoietin receptor MPL are less frequent, and account for only 5–8% of ET and PMF patients. The most common mutation at position 515 is located in the juxtamembrane protein region and induces constitutive activation of MPL and, consecutively, JAK2 signaling [8]. Several missense mutations, including W515L and W515K, and, rarely, other mutations have also been reported and analogously induce MPL activation [9]. Thus, it has become clear that driver mutations in JAK2, CALR, and MPL converge on the activation of JAK2 signaling as a common feature [43].

### 2.2. Triple-Negative MPN

For 10–15% of PMF and ET patients, neither *JAK2*V617F nor a CALR or MPL driver mutation are identified. These patients, who are termed “triple-negative” driver mutations, may be found later in the clinical course or upon genetic testing with higher sensitivity. Non-canonical mutations in *JAK2* (e.g., V625F, F556V, R683G, and E627A) or *MPL* (e.g., S505N, S204P, T119I, and Y591D/N) were found in some triple-negative patients and shown to result in constitutive activation of the JAK-STAT signaling [44,45,46]. Some of these mutations are germline rather than somatically acquired genetic alterations, therefore representing a familial, non-clonal erythrocytosis or thrombocytosis. In the remaining proportion of ET and PMF patients, a driver mutation cannot be identified, suggesting an unknown genetic alteration underlying MPN pathogenesis or that they might not actually have a malignancy.

### 2.3. Concomitant Gene Mutations in Myeloid Cancer Genes

The advent of modern sequencing technologies including next generation sequencing (NGS) has enabled a detailed characterization of the genetic/genomic landscape of MPN in recent years. These comprehensive investigations of genetic alterations underlying MPN have revealed that additional somatic mutations co-occur with the driver mutations in JAK2, CALR, and MPL genes in more than half of MPN patients [11,33,44]. They mostly affect genes commonly mutated in myeloid malignancies include acute myeloid leukemia (AML) and myelodysplastic syndrome (MDS). They primarily affect epigenetic regulators, factors of the mRNA splicing machinery, and signaling molecules, while a set of specific mutations is indicative of imminent leukemic transformation. The overall number of somatic mutations is higher in PMF as compared to PV and ET and has been shown to confer an adverse prognostic effect [11,33,44].

Epigenetic regulators. Mutations in epigenetic regulators include *TET2* mutations in 10–15%, *DNMT3A* and *ASXL1* mutations in 5–15%, *EZH2* in 3–10%, and *IDH1/2* mutations in 1–2% of MPN patients. *TET2* mutations impair TET2 enzymatic function, which catalyzes the conversion of 5-methylcytosine (5-mc) to 5-hydroxymethylcytosine (5-hmc) and initiates DNA demethylation and upregulated transcription. It has been shown in murine models that *TET2* mutations promote the expansion of the hematopoietic stem cell pool [47,48,49], but their prognostic significance regarding transformation and survival is unclear [42,43]. The order of appearance of TET2 and JAK2 mutations impacts on the clinical phenotype, with “JAK2 first” patients typically presenting with PV, while “TET2 first” patients would rather present with ET/PMF [32]. TET2 function is modulated by the conversion of isocitrate to alpha-ketoglutarate catalyzed by isocitrate dehydrogenases IDH1 and IDH2. *IDH1/2* mutations interfere with this process and lead to the accumulation of the oncometabolite 2-hydroxyglutarate impeding TET2 function [50]. *IDH1/2* mutations confer adverse prognostic effects in MPN and are enriched in patients progressing to secondary AML [51].

Analysis of global DNA methylation patterns in MPN patients identified differential methylation as compared to healthy controls highlighting the important role of perturbed DNA methylation in MPN [52]. DNA methyltransferases as DNMT3A mediate de novo DNA methylation and DNMT3A mutations were found to be prevalent across myeloid malignancies, primarily in AML and MPN [53]. The R882H mutation is most frequent and mediates DNMT3A loss of function leading to expansion of the hematopoietic stem cell pool and increased gene transcription and thrombocytosis in murine models [54,55]. *DNMT3A* mutations are considered early events in MPN evolution and, similarly to *TET2* mutations, the order of acquisition affects the MPN phenotypes with a propensity for ET upon DNMT3A followed by *JAK2* mutations and for PV upon acquisition of DNMT3A after *JAK2* mutations [56].

Mutations in EZH2 and ASXL1 interfere with the function of the polycomb repressive complex 2 (PRC2), which mediates histone modification via histone H3 di- and tri-methylation at lysine 27 (H3K27me2/3). *ASXL1* mutations frequently occur in PMF patients (10–35%) and are rather rare in ET or PV (2–5%). As in other myeloid malignancies, they confer an adverse prognosis with inferior survival [57,58,59]. EZH2, which represents the PRC2 catalytic component, is mostly mutated in MF patients (2–10%) and is associated with an unfavorable prognosis [60,61]. The JAK2V617F mutation also interferes with epigenetic processes, in addition to mediating constitutive activation of hematopoietic cytokine signaling. It has been shown that both wild-type and mutant JAK2 translocate to the nucleus and phosphorylate arginine methyltransferase PRMT5, which highlights the importance of histone methylation changes in MPN [62,63,64].

Splicing factors. Mutations in RNA splicing factor genes, including *SRSF2*, *SF3B1*, *U2AF1*, and *ZRSR2*, which are characteristic of myelodysplastic syndromes, are also prevalent in PMF (5–15%), but rare in ET and PV (1–5%). They lead to perturbation of mRNA splicing [65]. For *SRSF2* mutations, an unfavorable prognosis with lower overall survival has been observed. Genetic alterations in SRSF2 are enriched in secondary AML, similarly to IDH1/2 and TP53 mutations [66].

## 3. Genetic Testing in Clinical Settings in MPN

**Figure 2:**
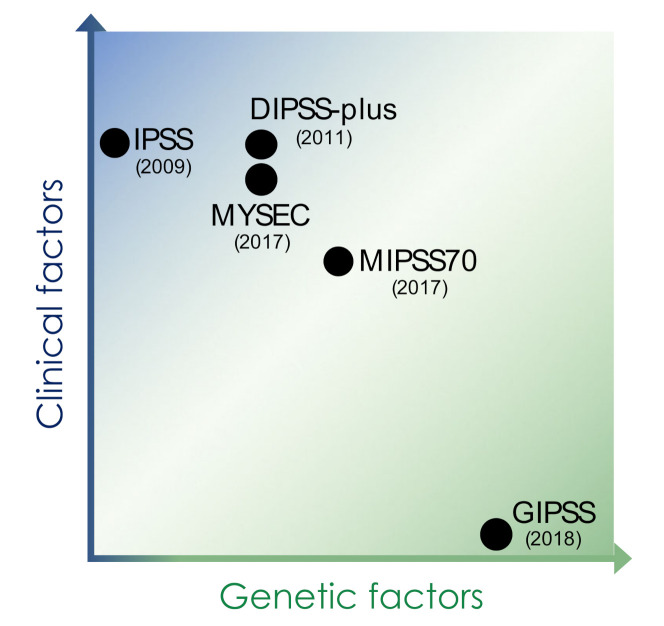
Prognostic scoring systems for myelofibrosis. Early prognostication schemes for myelofibrosis relied on clinical factors including age, hematologic parameters including cytopenias and blast count, and symptoms, while more recent scores have increasingly implemented genetic factors such as information on cytogenetic features, driver mutations, and concomitant somatic mutations of prognostic impact. IPSS: international prognostic scoring system, DIPSS: dynamic international prognostic system, MYSEC: myelofibrosis secondary to PV and ET-prognostic model, MIPPS70: mutation-enhanced international prognostic score system (validated up to 70 years of age), GIPSS: genetically inspired prognostic scoring system.

Modern sequencing efforts over the last decade have revealed the heterogeneous genetic landscape of MPN in large parts. These discoveries into the genetic basis of MPN have led to a detailed insight into disease development, clonal evolution, and progression of MPN and have revealed specific molecular features of clinical sub-entities such as PV, ET, and MF. This genetic knowledge is increasingly integrated into clinical practice including the implementation into standard diagnostic criteria and modern prognostication schemes (Table 1, Figure 2) [19,67].

### 3.1. Genetic Testing for MPN Diagnosis

Based on the extensive genetic characterization of MPN over the last years, driver mutations can be detected by routine genetic testing in 98% of PV patients and 85–90% of ET and PMF patients. The presence of a *JAK2*, *CALR*, or *MPL* driver mutation is not specific, but highly suggestive of an MPN and thus represents a valuable tool in support of diagnosing PV, ET, or PMF [68]. Accordingly, *JAK2*, *CALR*, or *MPL* driver mutations have been implemented as a major criterion into the standard diagnostic criteria by the World Health Organization (WHO) for PV, ET, and PMF [15]. The ease and high reproducibility of genetic testing has supported this process, while other, more laborious and delicate assays such as the evaluation of endogenous colony formation have been largely abandoned [15]. The extensive availability of next generation sequencing platforms at tertiary care centers has further supported the testing for additional gene mutations prevalent in MPN. This is particularly helpful to demonstrate clonality of hematopoiesis in triple negative MPN patients, and such testing is recommended by the WHO 2016 guidelines for *ASXL1*, *EZH2*, *IDH1/2*, *SRSF2*, *TET2*, and *SF3B1* in the absence of the classical driver mutations [19]. Despite the fulminant development of genetic/genomic tools for routine diagnostics, conventional cyto- and histomorphologic assessment remains valuable given the long-standing experience with these readouts and their availability in less-equipped settings.

### 3.2. Genetic Testing for Prognostication and Treatment Decisions

Correlative studies have revealed associations of specific gene mutations with clinical presentation, progression dynamics and outcome, and this knowledge has been increasingly utilized for prognostication of individual patients’ courses. It has become clear that *CALR* mutations are associated with lower risk courses in terms of thrombosis and leukemic transformation, with the longest overall survival in CALR mutated and ASXL1 unmutated patients [69,70]. In contrast, it has consistently been shown that the presence of the so-called “high molecular risk” (HMR) mutations affecting *ASXL1*, *EZH2*, *IDH1/2* and *SRSF2* relates to adverse prognosis [12,66].

While prognostic modeling for PMF relied on the international prognostic scoring system (IPSS) in 2009 [71], which is solely based on age and hematological parameters for risk stratification, more recent schemas have implemented molecular factors to refine prognostication (Table 1, Figure 2). Thus, the dynamic IPSS-plus score (DIPSSplus) involves the patient’s cytogenetic features, while the MYSEC score specifically validated for post-ET and post-PV MF considers the presence or absence of CALR mutations [13,72]. The mutation enhanced IPSS-70 (MIPSS70), which was specifically developed for assisting the decision making for allogeneic stem cell transplantation in MF patients up to 70 years of age, involves CALR mutation status as well as the presence and number of HMR mutations including ASXL1, EZH2, IDH1/2, and SRSF2 mutations [20]. It was further refined by adding cytogenetic information including unfavorable and very high risk karyotypes as well as U2AF1 mutations in the MIPSS70-plus 2.0 [73]. The genetically inspired IPSS (GIPSS) so far represents the only prognostication scheme purely relying on genetic factors including driver mutations, HMR mutations, and cytogenetic information [65]. Given the significance of molecular factors for the prognosis of MF patients, genetic features have a growing impact also on therapeutic decisions. Currently, the European Leukemia Net (ELN) and the European Society on Blood and Marrow Transplantation (EBMT) advise to evaluate MF patients for allogeneic hematopoietic stem cell transplantation if their IPSS or DIPSS scores reach intermediate-2 or high risk or if they show intermediate-1 risk disease with molecular high risk features such as *ASXL1* mutations or adverse cytogenetics [74].

For PV and ET patients, thrombotic and hemorrhagic events represent the major cause of morbidity and mortality, but fibrotic or leukemic transformation may also occur. Current prediction tools, such as the international prognostic score for thrombosis in ET (IPSET-thrombosis), focus on the risk for thrombo-hemorrhagic complications and rely on clinically derived variables including age, previous thrombosis, and presence of cardiovascular risk factors and the *JAK2*V617F mutation [75]. Although additional somatic mutations in myeloid cancer genes are less frequent in ET and PV than in MF, recent studies have shown that genetic information could also support outcome prediction in ET and PV. This has led to the development of MIPSS scores for ET and PV, which implement the information on spliceosome mutations, particularly SRSF2, SF3B1, and U2AF1 mutations, in addition to the established clinical factors age, leukocyte counts, and history of thrombosis [76].

Currently, further detailed prognostication tools are being developed, which aim for personalized predictions based on large scale genomic analysis [44] (https://cancer.sanger.ac.uk/mpn-multistage, accessed on 31 July 2021). These developments will hopefully soon reach clinical practice to support specifically tailored therapeutic approaches for individual patients.

## 4. Molecular Therapies in MPN

### 4.1. Treatment Recommendation

**Figure 3:**
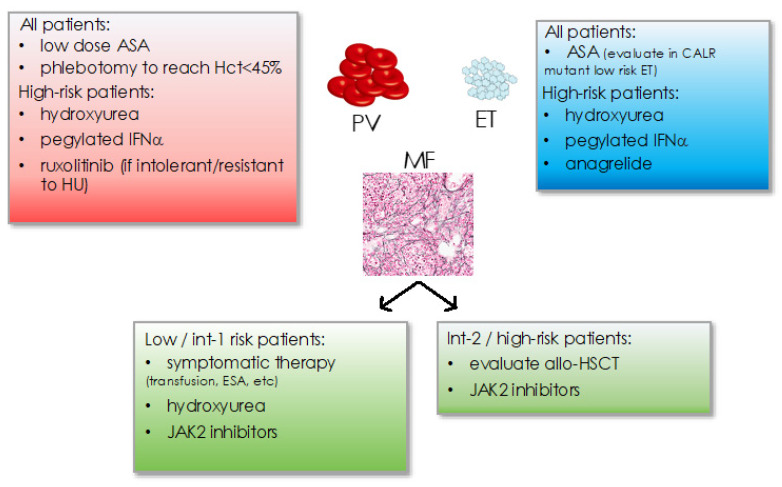
Current treatment recommendations in MPN for PV, ET, and MF subtypes. Hct: hematocrit, ASA: acetyl-salicylic acid, IFNα: interferon-alpha, int: intemediate, ESA: erythropoiesis stimulating agents, allo-HSCT: allogeneic hematopoietic stem cell transplantation.

Classic treatment of MPN has traditionally focused on platelet inhibition as well as cytoreductive measures including phlebotomies to achieve hematocrit <45% in PV or hydroxyurea, anagrelide, or pegylated interferon-alpha (Figure 3) [19,77]. Cytoreductive treatment is recommended in patients aged >60 years or with a history of thrombosis or excessive thrombocytosis to counteract an increased risk for thrombo-hemorrhagic complications. Management of symptoms such as cytopenias (e.g., by transfusions, erythropoietin substitution, etc.) or splenomegaly is essential in myelofibrosis. Intermediate-2 to high risk myelofibrosis or the presence of high risk molecular features mandates the evaluation of allogeneic hematopoietic stem cell transplantation as the only curative treatment concept so far.

### 4.2. JAK2 Inhibitors

The increasing insight into the molecular pathogenesis of MPN has revealed rational targets for therapy. The discovery of the *JAK2*V617F oncogene and the finding that CALR and MPL mutations similarly activate JAK2 signaling has posed the JAK2 tyrosine kinase as an important therapeutic target [78]. The JAK1/2 inhibitor ruxolitinib approved in 2012 represents a current standard of care for the treatment of symptomatic intermediate to high risk MF and for PV patients intolerant or refractory to hydroxyurea [21,22,23]. Fedratinib, a JAK2/FLT3 inhibitor with analogous type 1 mode of JAK2 binding, has recently been approved for MF patients [24]. Neurologic toxicities, which became evident in the JAKARTA trials including Wernicke’s encephalopathy were judged as rare and manageable by preventive measures such as thiamine supplementation and monitoring [79]. Additional JAK2 inhibitors are currently in advanced clinical trials and include momelotinib and pacritinib. They show promising profiles for anemic and thrombocytopenic MF patients and are thus eagerly awaited [80,81,82]. JAK2 inhibitors such as ruxolitinib provide clinical benefits in terms of splenomegaly reduction and symptom control and have demonstrated a survival benefit [21,22,83]. However, their disease-modifying effects are modest, with a limited potential for mutant clone reduction and continuing clonal evolution [84,85]. Refined JAK2 inhibitors truly reducing the MPN clone and halting clonal progression are highly desirable. Investigative efforts are ongoing, as, e.g., for JAK inhibitors with a type II mode of binding similar to BCR-ABL inhibitors [86,87]. In addition, mutant calreticulin, which is exposed at the cell surface in association with MPL, could be addressed as an therapeutic target, directly relating to JAK2-STAT signaling in CALR mutant patients [38,88,89].

### 4.3. Resistance to JAK Inhibitors

The limited clinical efficacy of JAK2 inhibitors as ruxolitinib seen in treatment-naïve patients, represents an active area of research. It has been shown that the MAPK pathway involving MEK and ERK kinases, remains activated despite JAK2 inhibitor therapy and requires to be targeted to increase therapeutic efficacy [90,91]. Several mechanisms have been reported including compensatory PDGF-PDGFR signaling or nuclear translocation of the splicing factor YBX1 ultimately resulting in persistent activation of the MAPK pathway [90,92].

In addition, clinical responses to the treatment with the JAK2 inhibitor ruxolitinib are lost in a substantial proportion of patients, as reflected by loss of the spleen response in approximately half of the patients with initial therapeutic benefit over five years [83]. Secondary mutations inducing genetic resistance as seen in BCR-ABL driven malignancies exposed to BCR-ABL inhibitors occur upon exposure to JAK2 inhibitors in vitro, but have not been observed in MPN patients [93,94]. In contrast, adaptive changes of JAK2 signaling have been described, which are reversible. It has been shown that JAK2 is able to form heterodimers with JAK1 or TYK2 upon JAK2 inhibitor treatment, which leads to reactivation of JAK2 signaling and re-occurrence of symptoms [87,95]. Of note, MPN cells would re-sensitize to JAK inhibitor therapy upon cessation and re-exposure, a phenomenon which has also been observed in patients [96]. Additional mechanisms of resistance have also been described such as pro-survival signals emerging from hematopoietic and/or stromal cells of the bone marrow microenvironment. Aberrant pro-inflammatory cytokine signals from both JAK2 mutant and non-mutant bone marrow cells have also been implicated as mediators of resistance to JAK2 inhibition [97,98].

### 4.4. Novel Therapies in MPN

Given the limitations of the current targeted therapies with JAK2 inhibitors, there is an important need for improved therapeutic approaches with enhanced clinical efficacy. Targeting JAK2 currently remains a central aspect of MPN treatment given the essential role of aberrant JAK2 signaling in PV, ET, and MF. Dual treatment approaches combining JAK2 inhibitors with additional therapeutic principles are thus being explored as promising options. Clinical studies are currently evaluating approaches of JAK2 inhibition in combination with inhibition of MEK-ERK or PI3K signaling, interference with apoptosis regulation by Bcl-2/Bcl-xL inhibition such as navitoclax, targeting methylation changes by the hypomethylating agents azacytidine or decitabine, as well as by histone deacetylase inhibitors panobinostat or givinostat [90,99,100,101,102]. As epigenetic mechanisms enhance the inflammatory milieu in MPN via activation of NF-kB signaling, BET inhibition has gained important interest as a therapeutic approach in MPN. A phase 2 study with the BET inhibitor CPI-0610 as monotherapy or in combination with ruxolitinib is currently ongoing in MF patients [103,104]. Of note, immune modulatory effects by interferon alpha represent a longstanding treatment concept in MPN, which has recently gained substantial interest as novel pegylated forms of interferon alpha with improved tolerability profiles have become available [105]. Pegylated interferon alpha has evident potential to decrease MPN clone size and has been approved for the treatment of PV [105], while data in MF is accumulating [106,107]. Pegylated interferon alpha is also a promising approach in combination with JAK2 inhibition by ruxolitinib, as evidenced by recent/ongoing clinical studies leading to decreased mutant allele burden and lowered symptom burden [108,109].

Several additional innovative treatment approaches are under clinical investigation, such as telomerase inhibition interfering with telomere function, MDM2 inhibition impacting on TP53 tumor suppressor function, and others [110]. These efforts will hopefully soon add valid options to the molecularly targeted treatment approaches for MPN patients.

## 5. Perspectives

The advent of modern sequencing technologies has driven the progress in the molecular understanding of MPN pathogenesis. These insights into the genetic/genomic landscape of MPN has translated in recent years to the routine clinical work-up of MPN patients integrating molecular markers into the diagnostic criteria and providing molecularly enriched prognostication schemes as a basis for decisions on clinical management. It is a hopeful perspective that additional, novel omics technologies addressing, e.g., the methylome and/or spliceosome of MPN will also be available for molecular diagnostics of MPN in the future and will inform further advanced, molecular-based therapeutic approaches for MPN patients.

## Figures and Tables

**Table 1:** Risk stratification schemes for myelofibrosis involving clinical and genetic factors.

	IPSS	DIPSS-Plus	MIPSS70	MYSEC	GIPSS
Genetic factors	−	unfavorable karyotype (+8, −7/7q−, i(17q), inv(3), −5/5q−, 12p− or 11q23)	absence of **CALR** type 1 mutations presence of 1 or more HMR mutations (**ASXL1, EZH2, SRSF2, IDH1**/**2**)	absence of **CALR** mutations	absence of **CALR** type 1/like mutations presence of **ASXL1, SRSF2, U2AF1**Q157 mutations unfavorable karyotype
Clinical factors	age >65 hemoglobin <100 g/L WBC > 25 × 10^9^/L circulating blasts ≥1% constitutional symptoms	age >65 hemoglobin <100 g/L WBC > 25 × 10^9^/L circulating blasts ≥1% constitutional symptoms need for RBC transfusion platelets < 100 × 10^9^/L	hemoglobin <100 g/L WBC > 25 × 10^9^/L platelets < 100 × 10^9^/L circulating blasts ≥2% fibrosis grade ≥2 constitutional symptoms	age at diagnosis hemoglobin <100 g/L platelets < 150 × 10^9^/L circulating blasts ≥3% constitutional symptoms	−
Risk category (points)	low (0) intermediate-1 (1) intermediate-2 (2) high (3)	low (0) intermediate-1 (1) intermediate-2 (2–3) high (≥4)	low (0–1) intermediate (2–4) high (≥5)	low (0) intermediate-1 (1) intermediate-2 (2) high (≥3)	low (0) intermediate-1 (1) intermediate-2 (2) high (≥3)
Target patients	MF patients at diagnosis	MF patients at diagnosis and at any time point during clinical course	MF patients at evaluation for allogeneic HSCT	post-ET and post-PV myelofibrosis patients	PMF patients

IPSS: international prognostic scoring system, DIPSS: dynamic international prognostic system, MYSEC: myelofibrosis secondary to PV and ET-prognostic model, MIPPS70: mutation-enhanced international prognostic score system (validated up to 70 years of age), GIPSS: genetically inspired prognostic scoring system.
